# Risk factors for positive urine culture and antimicrobial resistance in suspected UTI after flexible ureteroscopic lithotripsy

**DOI:** 10.3389/fsurg.2026.1845237

**Published:** 2026-05-07

**Authors:** Hongbo Wang, Wenzhi Gao

**Affiliations:** 1 Department of Emergency, Peking University First Hospital, Beijing, China; 2 Department of Urology, Peking University First Hospital-Miyun Hospital, Beijing, China

**Keywords:** antimicrobial resistance, flexible ureteroscopic lithotripsy, pseudomonas aeruginosa, risk factors, urinary tract infection, urine culture

## Abstract

**Background:**

This study identified independent risk factors for positive urine culture and bacterial resistance in patients with suspected urinary tract infection (UTI) after flexible ureteroscopic lithotripsy (FURL), to provide an evidence-based basis for individualized clinical antimicrobial treatment strategies.

**Methods:**

A retrospective cohort study enrolled 864 adult patients with suspected UTI after FURL who underwent urine culture between January 2024 and June 2025. Baseline clinical data were collected, and univariate and multivariate logistic regression analyses were used to identify independent risk factors for positive urine culture, pseudomonas aeruginosa infection, and bacterial resistance.

**Results:**

Univariate and multivariate logistic regression analysis demonstrated that advanced age, female gender, positive urine nitrite, positive urine glucose, urinary catheterization, hydronephrosis, and ureteral stricture were independent risk factors for positive urine culture in patients with suspected UTI after FURL. Young age and elevated urinary nitrite were identified as independent risk factors for Pseudomonas aeruginosa infection in patients with positive urine culture. Additionally, female gender was an independent risk factor for antimicrobial resistance in patients with positive urine culture, while diabetes mellitus was an independent protective factor for antimicrobial resistance with positive urine culture.

**Conclusion:**

Distinct high-risk factors correlate with positive urine culture, P. aeruginosa infection and antimicrobial resistance in suspected UTI patients after FURL; stratified clinical assessment by these factors enables individualized antimicrobial therapy to improve treatment precision, reduce irrational antibiotic use and alleviate resistance development.

## 1 Introduction

Urinary tract infection (UTI) is one of the most prevalent bacterial infectious diseases in clinical practice. Empirical antibiotic use, inappropriate dosing, and delayed clinical interventions can easily induce severe complications such as pyelonephritis and sepsis, posing a serious threat to patient health (1–3). Although the clinical application of antibiotics has markedly reduced the incidence of sepsis, the extensive and inappropriate use of these agents not only increases the risk of adverse drug reactions but also drives the continuous escalation of bacterial resistance (4–6). Currently, antimicrobial resistance has emerged as a major global public health challenge with the widespread use of antibiotics (7).

Empirical antimicrobial therapy for patients with suspected UTI after flexible ureteroscopic lithotripsy (FURL) fails to benefit some individuals and may even expose them to medication-related adverse effects (8, 9). Thus, developing individualized medication strategies for patients with suspected UTI after FURL presenting different clinical characteristics is critical. Clarifying the high-risk features of positive urine culture in this population can guide the rational selection of clinical antibiotics. Further, exploring the high-risk factors for bacterial resistance can facilitate more prudent decision-making regarding antimicrobial use in high-risk patients, thereby optimizing clinical treatment regimens.

In this study, we performed a risk factor analysis with positive urine culture and antimicrobial susceptibility test resistance as the primary outcomes in patients with suspected UTI after FURL. The primary objective was to identify high-risk populations for positive urine culture and bacterial resistance, providing evidence-based references for the formulation of individualized clinical antimicrobial treatment strategies.

## 2 Methods

**Figure 1:**
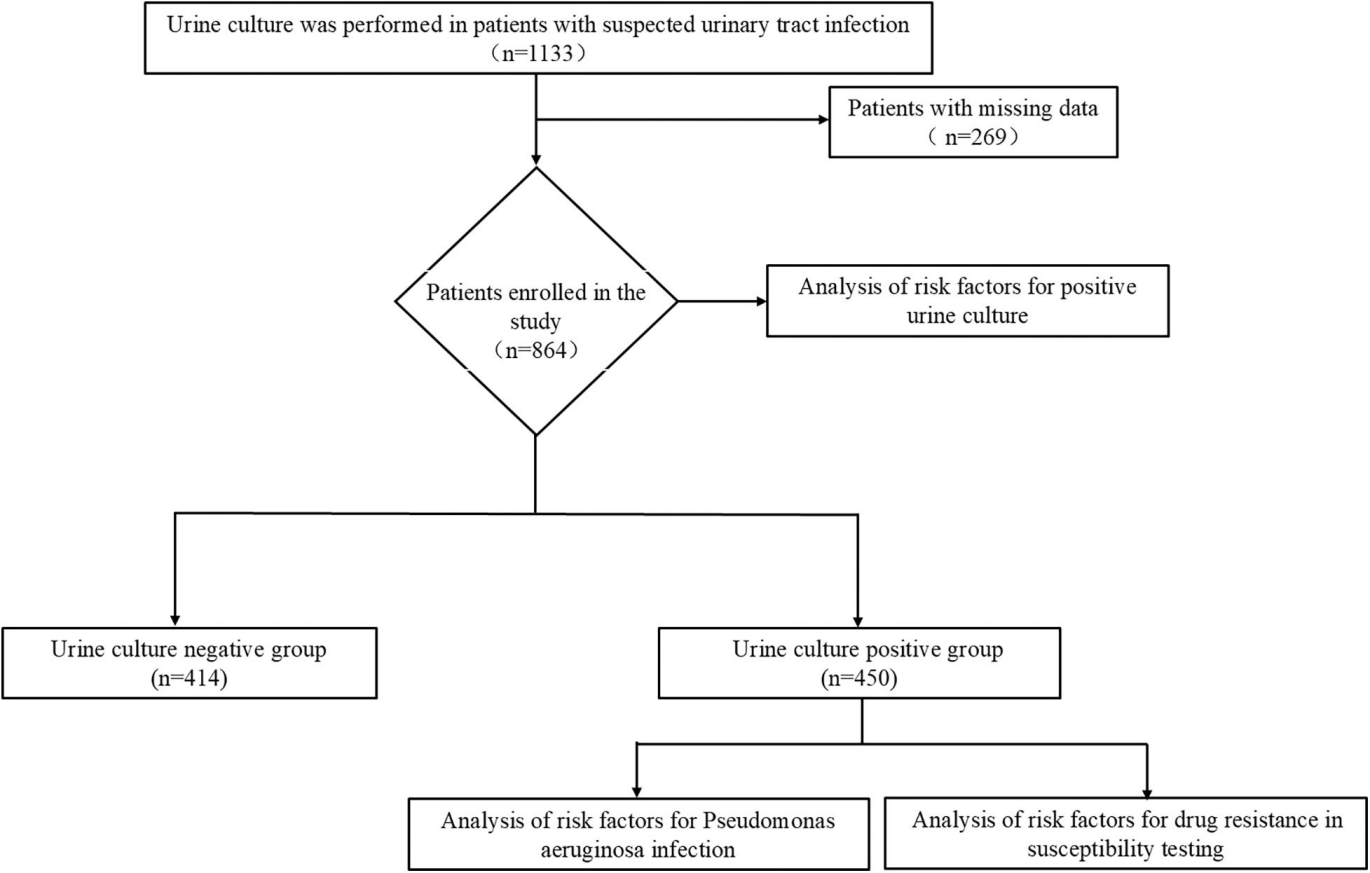
Study flow chart.

A total of 1,133 patients with suspected UTI after FURL underwent urine culture at Peking University First Hospital, Beijing Miyun District Hospital, Beijing Jiangong Hospital, Fuxing Hospital Affiliated to Capital Medical University, and Yuquan Hospital Affiliated to Capital Medical University from January 2024 to June 2025. A total of 250 patients were excluded due to missing data, and 864 patients with complete baseline data were enrolled in the final analysis, including 450 with positive urine culture and 414 with negative urine culture (Figure 1). In this study, FURL was uniformly performed for the treatment of renal and ureteral calculi, and all procedures were completed using single-use flexible ureteroscopes (Promisemed, VeriEndo®).

In this study, patients with suspected UTI after FURL were defined as those who presented with local urinary tract signs/symptoms, fever, or chills within 3 months postoperatively (10–13). A positive urine culture was defined as a clean midstream urine colony count of ≥10^5^ colony-forming units per milliliter (CFU/mL) (14). Urinary catheterization included indwelling urethral catheterization and percutaneous nephrostomy, with the latter primarily applied in patients with poor renal function (elevated serum creatinine levels) or severe hydronephrosis. Residual calculi were defined as the presence of stones >3 mm in diameter detected by ultrasonography, intravenous urography, or computed tomography within 1 week after the completion of surgery (15, 16). In this study, antimicrobial resistance was defined based on single-drug resistance, i.e., a strain was considered resistant if it exhibited resistance to any one of the antibiotics within the test panel (17).

Inclusion criteria: (1) Aged ≥18 years; (2) Within 3 months post-FURL; (3) Clinical suspicion of UTI; (4) Underwent urine culture testing; (5) Complete clinical and laboratory data available; (6) Negative urine culture prior to FURS. Exclusion criteria: (1) Diagnosis of immune-related diseases or receipt of immunosuppressant therapy. (2) History of psychiatric disorders.

The clinical variables included in the study were gender, age, body mass index, diabetes mellitus, hypertension, coronary heart disease, diameter of the calculus, operation time, postoperative residual calculus, postoperative indwelling double-J ureteral stent, urine leukocytes, urine protein, urine nitrite, urinary catheterization, hydronephrosis, urogenital tumor, ureteral stricture, and preoperative antibiotic use. With positive urine culture, Pseudomonas aeruginosa infection, and antimicrobial susceptibility test resistance as the primary outcomes, we aimed to explore the independent risk factors for positive urine culture in patients with suspected UTI after FURL and further analyze the independent risk factors for Pseudomonas aeruginosa infection and antimicrobial resistance in those with positive urine culture.

This study was conducted in accordance with the principles of the Declaration of Helsinki (revised 2013) and was approved by the Ethics Committee of Peking University First Hospital. Informed consent was waived for this retrospective analysis due to its non-interventional nature.

## 3 Results

### 3.1 Risk factors for positive urine culture in patients with suspected UTI after FURL

**Figure 2:**
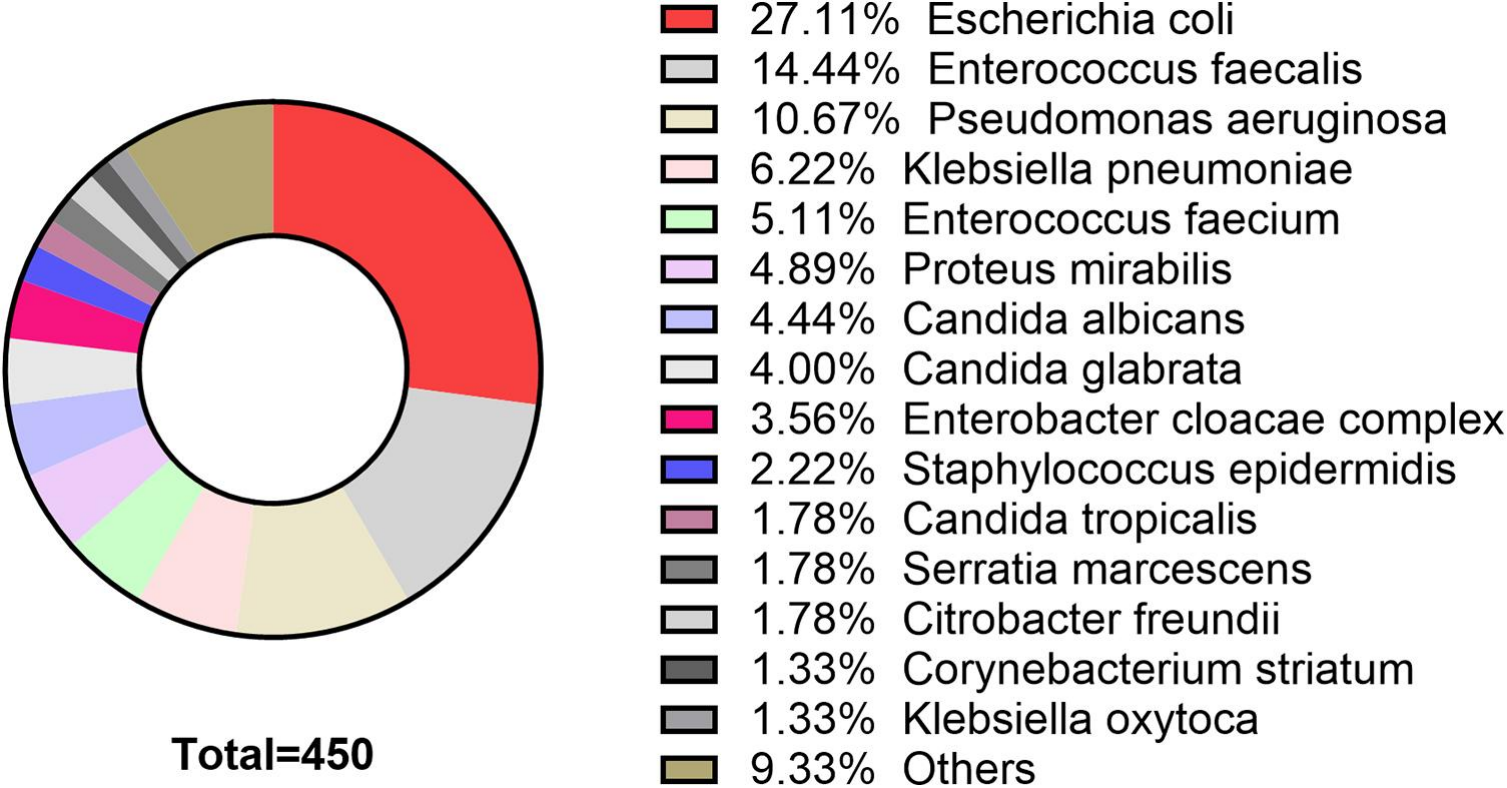
Pathogen distribution in patients with positive urine culture.

Baseline clinical characteristics of the positive urine culture group and negative urine culture group are summarized in Table 1. Statistically significant differences were observed between the two groups in age (*P* < 0.001), BMI (*P* = 0.021), gender (*P* < 0.001), urine glucose status (*P* < 0.001), urine nitrite status (*P* < 0.001), urinary catheterization (*P* < 0.001), hydronephrosis (*P* < 0.001), and ureteral stricture (*P* < 0.001). The distribution of pathogenic bacteria in patients with positive urine culture is presented in Figure 2. No patients in this study developed sepsis.

**Table 1:** Comparison between urine culture negative group and urine culture positive group.

Variable	Total	Urine culture negative group	Urine culture positive group	*P* value
Total number of patients	864	414	450	
Age (years)	56.50 ± 15.00	54.36 ± 14.26	58.48 ± 15.44	<0.001
BMI (kg/m^2^)	25.17 ± 3.83	25.48 ± 3.58	24.87 ± 4.04	0.021
Gender, *n*%				<0.001
Male	523 (60.53)	315 (76.09)	208 (46.22)	
Female	341 (39.47)	99 (23.91)	242 (53.78)	
Hypertension, *n*%				0.061
Yes	286 (33.10)	150 (36.23)	136 (30.22)	
No	578 (66.90)	264 (63.77)	314 (69.78)	
Diabetes mellitus, *n*%				0.307
Yes	216 (25.00)	97 (23.43)	119 (26.44)	
No	648 (75.00)	317 (76.57)	331 (73.56)	
Coronary heart disease, *n*%				0.881
Yes	45 (5.21)	21 (5.07)	24 (5.33)	
No	819 (94.79)	393 (94.93)	426 (94.67)	
Diameter of the calculus (cm)	2.04 ± 0.84	2.08 ± 0.85	2.01 ± 0.85	0.289
Operation time (min)	67.18 ± 30.94	66.78 ± 31.00	67.56 ± 30.99	
Postoperative residual calculus, *n*%				0.815
Yes	22 (2.55)	10 (2.42)	12 (2.67)	
No	842 (97.45)	404 (97.58)	438 (97.33)	
Postoperative indwelling double-J ureteral stent, *n*%				0.652
Yes	538 (62.27)	261 (63.04)	277 (61.56)	
No	326 (37.73)	153 (36.96)	173 (38.44)	
Urinary protein, *n*%				0.531
Negative	529 (61.23)	249 (60.14)	280 (62.22)	
Positive	335 (38.77)	165 (39.86)	170 (37.78)	
Urinary glucose, *n*%				<0.001
Negative	757 (87.62)	383 (92.51)	374 (83.11)	
Positive	107 (12.38)	31 (7.49)	76 (16.89)	
Urinary nitrite, *n*%				<0.001
Negative	709 (82.06)	387 (93.48)	322 (71.56)	
Positive	155 (17.94)	27 (6.52)	128 (28.44)	
Catheterization, *n*%				<0.001
Yes	281 (32.52)	87 (21.01)	194 (43.11)	
No	583 (67.48)	327 (78.99)	256 (56.89)	
Hydronephrosis, *n*%				<0.001
Yes	148 (17.13)	42 (10.14)	106 (23.56)	
No	716 (82.87)	372 (89.86)	344 (76.44)	
Urogenital tumor, *n*%				0.919
Yes	34 (3.94)	16 (3.86)	18 (4.00)	
No	830 (96.06)	398 (96.14)	432 (96.00)	
Ureteral stricture, *n*%				<0.001
Yes	117 (13.54)	33 (7.97)	84 (18.67)	
No	747 (86.46)	381 (92.03)	366 (81.33)	
Preoperative antibiotic use, *n*%				0.085
Yes	169 (19.56)	91 (21.98)	78 (17.33)	
No	695 (80.44)	323 (78.02)	372 (82.67)	

BMI, body mass index.

Univariate and multivariate logistic regression analyses revealed that advanced age (*P* < 0.001), female gender (*P* < 0.001), positive urine nitrite (*P* < 0.001), positive urine glucose (*P* < 0.001), urinary catheterization (*P* < 0.001), hydronephrosis (*P* < 0.001), and ureteral stricture (*P* = 0.005) were independent risk factors for positive urine culture in patients with suspected UTI (Table 2).

**Table 2:** Analysis of risk factors for positive urine culture.

Characteristic	Univariate analysis		Multivariate analysis	
	OR (95% CI)	*P*-value	OR (95% CI)	*P*-value
Age	1.019 (1.010–1.028)	<0.001	1.022 (1.011–1.034)	<0.001
Gender	0.270 (0.202–0.362)	<0.001	0.387 (0.273–0.548)	<0.001
BMI	0.959 (0.926–0.994)	0.021	0.975 (0.933–1.020)	0.273
Hypertension	0.762 (0.574–1.013)	0.061		
Diabetes mellitus	1.175 (0.862–1.601)	0.307		
Coronary heart disease	1.047 (0.573–1.912)	0.881		
Diameter of the calculus	0.918 (0.784–1.075)	0.289		
Operation time	1.001 (0.996–1.005)	0.716		
Postoperative residual calculus	0.903 (0.386–2.114)	0.815		
Postoperative indwelling double-J	0.939 (0.713–1.236)	0.652		
Urinary protein	1.091 (0.830–1.435)	0.531		
Urinary nitrite	5.698 (3.667–8.852)	<0.001	3.954 (2.419–6.460)	<0.001
Urinary glucose	2.511 (1.615–3.903)	<0.001	2.867 (1.714–4.795)	<0.001
Catheterization	2.848 (2.108–3.849)	<0.001	2.220 (1.541–3.198)	<0.001
Hydronephrosis	2.729 (1.855–4.016)	<0.001	2.752 (1.725–4.389)	<0.001
Ureteral stricture	2.650 (1.728–4.063)	<0.001	2.256 (1.281–3.976)	0.005
Urogenital tumor	1.036 (0.521–2.060)	0.919		
Preoperative antibiotic use	0.744 (0.531–1.043)	0.086		

### 3.2 Risk factors for Pseudomonas aeruginosa infection in patients with positive urine culture

The clinical characteristics of patients with positive urine culture stratified by Pseudomonas aeruginosa infection status are shown in Supplementary Table S1.

Univariate and multivariate logistic regression analyses indicated that young age (*P* = 0.003) and urinary nitrite (*P* = 0.002) were independent risk factors for Pseudomonas aeruginosa infection in patients with positive urine culture (Table 3).

**Table 3:** Analysis of risk factors for Pseudomonas aeruginosa infection in patients with positive urine culture.

Characteristic	Univariate analysis		Multivariate analysis	
	OR (95% CI)	*P*-value	OR (95% CI)	*P*-value
Age	0.958 (0.938–0.979)	<0.001	0.964 (0.942–0.988)	0.003
Gender	1.064 (0.563–2.010)	0.849		
BMI	0.992 (0.915–1.076)	0.854		
Hypertension	0.515 (0.232–1.143)	0.103		
Diabetes mellitus	0.435 (0.178–1.061)	0.067		
Urinary protein	0.640 (0.338–1.211)	0.170		
Diameter of the calculus	1.050 (0.721–1.530)	0.798		
Operation time	1.003 (0.992–1.013)	0.612		
Postoperative residual calculus	0.503 (0.106–2.374)	0.385		
Postoperative indwelling double-J ureteral stent	0.973 (0.433–2.186)	0.947		
Urinary nitrite	3.134 (1.645–5.972)	<0.001	2.899 (1.476–5.691)	0.002
Urinary glucose	0.226 (0.053–0.955)	0.043	0.433 (0.096–1.951)	0.276
Catheterization	1.223 (0.647–2.311)	0.536		
Hydronephrosis	2.174 (1.118–4.229)	0.022	1.678 (0.826–3.410)	0.152
Ureteral stricture	3.077 (1.567–6.043)	0.001	1.929 (0.946–3.932)	0.071
Urogenital tumor	1.225 (0.272–5.521)	0.792		
Preoperative antibiotic use	1.136 (0.504–2.559)	0.758		

### 3.3 Risk factors for antimicrobial resistance in patients with positive urine culture

Baseline clinical data of the non-antimicrobial resistant group and antimicrobial resistant group among patients with positive urine culture are detailed in Supplementary Table S2.

Univariate and multivariate logistic regression analyses confirmed that female gender (*P* < 0.001) was an independent risk factor for antimicrobial resistance in patients with positive urine culture, while diabetes mellitus (*P* = 0.013) was an independent protective factor for antimicrobial resistance in these patients (Table 4).

**Table 4:** Analysis of risk factors for antimicrobial resistance in susceptibility testing among patients with positive urine culture.

Characteristic	Univariate analysis		Multivariate analysis	
	OR (95% CI)	*P* value	OR (95% CI)	*P* value
Age	1.002 (0.989–1.016)	0.763		
Gender	0.387 (0.248–0.604)	<0.001	0.342 (0.213–0.550)	<0.001
BMI	0.967 (0.916–1.021)	0.231		
Hypertension	0.919 (0.581–1.452)	0.716		
Diabetes mellitus	0.495 (0.292–0.838)	0.009	0.500 (0.289–0.863)	0.013
Coronary heart disease	1.329 (0.553–3.192)	0.525		
Diameter of the calculus	0.886 (0.692–1.135)	0.340		
Operation time	1.001 (0.994–1.008)	0.743		
Postoperative residual calculus	1.983 (0.617–6.370)	0.250		
Postoperative indwelling double-J ureteral stent	1.025 (0.668–1.574)	0.910		
Urinary leukocytes	1.886 (1.155–3.079)	0.001	1.682 (0.951–2.975)	0.074
Urinary protein	0.815 (0.533–1.248)	0.347		
Urinary nitrite	1.667 (1.067–2.603)	0.025	1.405 (0.819–2.410)	0.217
Urinary glucose	0.748 (0.417–1.343)	0.331		
Catheterization	0.786 (0.514–1.203)	0.268		
Hydronephrosis	0.969 (0.592–1.586)	0.900		
Ureteral stricture	0.957 (0.559–1.639)	0.873		
Urogenital tumor	0.329 (0.074–1.452)	0.142		
Preoperative antibiotic use	1.083 (0.629–1.867)	0.773		

## 4 Discussion

UTI is a clinically prevalent bacterial infection, and the iatrogenic stimulation induced by FURL further increases the risk of infection, with the core challenge in its diagnosis and treatment lying in the precise regulation of empirical antimicrobial therapy (18). Rational antibiotic use effectively controls infection and reduces the risk of complications, whereas inappropriate use may result in the waste of medical resources, exacerbate bacterial resistance, and even trigger adverse drug reactions (19). Currently, clinical empirical antibiotic use for patients with suspected UTI lacks a targeted stratified basis: low-risk patients are often overtreated, while high-risk patients may experience poor infection control due to inappropriate medication (20). Therefore, identifying high-risk populations for positive urine culture, pathogenic bacterial infection, and bacterial resistance is of great clinical value for achieving individualized and precise antimicrobial treatment of UTI.

The results of this study identified age, gender, positive urine nitrite, positive urine glucose, urinary catheterization, hydronephrosis, and ureteral stricture as independent risk factors for positive urine culture in patients with suspected UTI after FURL. Rajanbir Kaur et al. (21) reported that sexual activity, gender, genetic factors, age, pregnancy-related factors, and indwelling urinary catheters are risk factors for complicated UTI, which is consistent with the findings of the present study. Advanced age is a key high-risk factor for positive urine culture. Elderly patients exhibit urinary tract mucosal atrophy, sphincter dysfunction, impaired urinary tract barrier function, and reduced systemic immunity. Additionally, they are more likely to have underlying diseases and urinary retention, leading to urine stasis that provides a favorable microenvironment for bacterial colonization and proliferation (11, 22). Age-related UTI susceptibility in women is closely associated with estrogen levels. Decreased estrogen promotes the shift of the urogenital flora from lactobacilli to Escherichia coli, thereby increasing infection risk. Women are inherently at a higher risk of UTI due to anatomical characteristics (a short, wide, and straight urethra) and the proximity of the urethral orifice to the anus and vagina, which facilitates the retrograde invasion of intestinal flora. Positive urinary nitrite is a direct biomarker of bacterial UTI, which reflects the proliferation of uropathogens to a certain extent and serves as an important predictor of positive urine culture (23). Positive urine glucose, typically associated with abnormal blood glucose levels, impairs urinary tract mucosal integrity and provides nutrients for bacterial growth, increasing the risk of true bacteriuria (24, 25). Urinary catheterization can damage the mucosal barrier, and bacterial biofilms readily form on catheter surfaces, inducing retrograde infection (26–30). Hydronephrosis and ureteral stricture cause impaired urinary drainage and urine stasis, leading to massive bacterial proliferation and renal dysfunction, which further increases the likelihood of positive urine culture (31). These risk factors compromise urinary tract defense function and alter bacterial colonization conditions from anatomical, physiological, and pathological perspectives. Clinically, they can be used as stratified screening indicators for positive urine culture in patients with suspected UTI, and standardized antimicrobial treatment evaluation should be prioritized for patients with multiple risk factors.

Pseudomonas aeruginosa is the primary pathogen responsible for refractory UTI, characterized by strong intrinsic resistance, easy acquisition of adaptive resistance, and biofilm formation. This pathogen is insensitive to many conventional antibiotics, and infection is prone to recurrence and refractoriness. Severe infections can induce life-threatening complications such as pyelonephritis and sepsis, associated with high treatment difficulty and poor prognosis (32, 33). In the present study, Age and elevated urinary nitrite were identified as independent risk factors for Pseudomonas aeruginosa infection in patients with positive urine culture. However, previous studies indicated that advanced age predisposes pseudomonas aeruginosa infection, which contradicts our findings (34, 35). This may be due to all enrolled patients having undergone ureteral lithotripsy. As an environmentally derived pathogen with strong adhesiveness that colonizes via minor urinary mucosal injuries, Pseudomonas aeruginosa is more prone to colonize young patients: their urinary mucosa with vigorous metabolism and abundant blood supply shows more pronounced inflammatory exudation after surgical microtrauma; additionally, higher ureteral smooth muscle tone in young patients makes submucosal microtears more likely under irrigation pressure, further expanding Pseudomonas aeruginosa colonization sites. Positive urinary nitrite may act as an independent risk factor for Pseudomonas aeruginosa infection for the following reasons: Pseudomonas aeruginosa can efficiently reduce urinary nitrate to nitrite in the microaerophilic urinary tract environment after FURL; nitrite enhances Pseudomonas aeruginosa virulence by upregulating its adhesive capacity and biofilm-forming ability; meanwhile, nitrite-mediated urinary alkalinization provides a favorable growth environment for pseudomonas aeruginosa. Furthermore, postoperative mucosal injury and nitrate accumulation further amplify this specific association (36). Identifying high-risk groups for Pseudomonas aeruginosa infection can guide clinical practice to implement targeted diagnosis and treatment in advance. For young patients with positive urinary nitrite after ureteral flexible lithotripsy, prophylactic use of anti-Pseudomonas aeruginosa sensitive antibiotics such as third-generation cephalosporins can improve infection control rates and reduce the risk of severe complications.

Studies from the United States and India have reported a sharp increase in the resistance of uropathogens to multiple clinically commonly used antibiotics, including penicillins and cephalosporins (37, 38). Against this backdrop, in-depth analysis of the clinical characteristics of patients with drug-resistant uropathogens is crucial for guiding rational antibiotic use and curbing the spread of drug-resistant strains. This study identified female gender as an independent risk factor for antimicrobial resistance in patients with positive urine culture, while diabetes mellitus was an independent protective factor against antimicrobial resistance in these patients. Female gender is a risk factor for antimicrobial resistance, likely due to the higher incidence and recurrence rate of UTI in women; repeated antimicrobial therapy and prolonged antibiotic exposure induce the development of adaptive resistance in uropathogens, increasing the rate of antimicrobial resistance (39, 40). In this study, diabetes mellitus was identified as an independent protective factor for antimicrobial resistance in patients with positive urine culture after FURL, which is contrary to the findings of conventional studies (41). The reason may be associated with the specific postoperative scenario of FURL: clinical perioperative management for diabetic patients is more rigorous, involving precise preoperative screening and standardized postoperative medication to avoid empirical use of antibiotics. Additionally, postoperative infections in diabetic patients are mostly caused by low-virulence strains, resulting in lower selective pressure for drug resistance, thus forming a reverse protective effect. Therefore, this study suggests that antibiotics should be selected more cautiously for female patients. For mild to moderate suspected infections, narrow-spectrum antibiotics targeting Gram-negative bacteria should be preferred. Meanwhile, the course of antibiotic therapy should be strictly controlled in accordance with the principle of short-course and adequate dosage to avoid unwarranted extension of medication.

This study provides an evidence-based reference for the precise clinical diagnosis and treatment of UTI but has several limitations that warrant further investigation. First, as a retrospective study, it is subject to selection bias. Additionally, detailed records of some clinical data (e.g., specific antibiotic types, treatment courses, and doses) were unavailable for some patients, which may have affected the analysis of risk factors for antimicrobial resistance. Second, the study population consisted of multi-center adult patients, and children and adolescents were not included, limiting the generalizability of the results and precluding the analysis of age-related differences in risk factors. Third, only common clinical variables were analyzed; potential influencing factors such as gene polymorphism, intestinal flora, and urine physicochemical indicators were not included, leading to an incomplete assessment of risk factors. Fourth, this was a cross-sectional study, and no long-term follow-up was performed, which prevented the analysis of the long-term effects of these risk factors on prognosis, recurrence rate, and antimicrobial resistance rate in patients with UTI.

## 5 Conclusion

Distinct high-risk factors correlate with positive urine culture, pseudomonas aeruginosa infection and antimicrobial resistance in suspected UTI patients after FURL; stratified clinical assessment by these factors enables individualized antimicrobial therapy to improve treatment precision, reduce irrational antibiotic use and alleviate resistance development.

## Data Availability

The raw data supporting the conclusions of this article will be made available by the authors, without undue reservation.
