# Supplementary material for: Risk factors for positive urine culture and antimicrobial resistance in suspected UTI after flexible ureteroscopic lithotripsy
Source: Front Surg. 2026 May 7;13:1845237. doi: 10.3389/fsurg.2026.1845237 (PMC13190483; doi:10.3389/fsurg.2026.1845237)
Supplement: Supplementary file 1 [file Supplementaryfile1.docx]

| Supplementary Table 1 Comparison between non-Pseudomonas aeruginosa positive group and Pseudomonas aeruginosa positive group in patients with positive urine culture. | | | |
| --- | --- | --- | --- |
| Variable | Non-Pseudomonas aeruginosa positive group | Pseudomonas aeruginosa positive group | P value |
| Number of patients | 408 | 42 |  |
| Age(years) | 59.44±15.10 | 49.24±15.84 | ＜0.001 |
| BMI (kg/m2) | 24.88±4.00 | 24.76±4.42 | 0.854 |
| Gender ,n％ |  |  | 0.849 |
| Male | 188（46.08） | 20（47.62） |  |
| Female | 220（53.92） | 22（52.38） |  |
| Hypertension, n％ |  |  | 0.098 |
| Yes | 128（31.37） | 8（19.05） |  |
| No | 280（68.63） | 34（80.95） |  |
| Diabetes mellitus, n％ |  |  | 0.061 |
| Yes | 113（27.70） | 6（14.29） |  |
| No | 295（72.30） | 36（85.71） |  |
| Coronary heart disease, n％ |  |  | 0.106 |
| Yes | 22（5.39） | 2（4.76） |  |
| No | 386（94.61） | 40（95.24） |  |
| Diameter of the calculus（cm） | 2.01±0.86 | 2.05±0.72 | 0.799 |
| Operation time（min） | 67.31±30.89 | 69.86±32.26 | 0.613 |
| Postoperative residual calculus, n％ |  |  | 0.376 |
| Yes | 10（2.45） | 2（4.76） |  |
| No | 398（97.55） | 40（95.24） |  |
| Postoperative indwelling double-J ureteral stent, n％ |  |  | 0.947 |
| Yes | 76（18.63） | 8（19.05） |  |
| No | 332（81.37） | 34（80.95） |  |
| Urinary protein ,n％ |  |  | 0.167 |
| Negative | 258（63.24） | 22（52.38） |  |
| Positive | 150（36.76） | 20（47.62） |  |
| Urinary glucose, n％ |  |  | 0.028 |
| Negative | 334（81.86） | 40（95.24） |  |
| Positive | 74（18.14） | 2（4.76） |  |
| Urinary nitrite, n％ |  |  | ＜0.001 |
| Negative | 302（74.02） | 20（47.62） |  |
| Positive | 106（25.98） | 22（52.38） |  |
| Catheterization,n％ |  |  | 0.536 |
| Yes | 174（42.65） | 20（47.62） |  |
| No | 234（57.35） | 22（52.38） |  |
| Hydronephrosis, n％ |  |  | 0.020 |
| Yes | 90（22.06） | 16（38.10） |  |
| No | 318（77.94） | 26（61.90） |  |
| Urogenital tumor, n％ |  |  | 0.791 |
| Yes | 16（3.92） | 2（4.76） |  |
| No | 392（92.08） | 40（95.24） |  |
| Ureteral stricture, n％ |  |  | ＜0.001 |
| Yes | 68（16.67） | 16（38.10） |  |
| No | 340（83.33） | 26（61.90） |  |
| Preoperative antibiotic use, n％ |  |  | 0.758 |
| Yes | 70（17.16） | 8（19.05） |  |
| No | 338（82.84） | 34（80.95） |  |
