# Supplementary material for: Risk factors for positive urine culture and antimicrobial resistance in suspected UTI after flexible ureteroscopic lithotripsy
Source: Front Surg. 2026 May 7;13:1845237. doi: 10.3389/fsurg.2026.1845237 (PMC13190483; doi:10.3389/fsurg.2026.1845237)
Supplement: Supplementary file 2 [file Supplementaryfile2.docx]

| Supplementary table 2 Comparison between antimicrobial resistant group and non-antimicrobial resistant group in patients with positive urine culture. | | | |
| --- | --- | --- | --- |
| Variable | Non-antimicrobial resistant group | Antimicrobial resistant group | P value |
| Number of patients | 329 | 121 |  |
| Age(years) | 58.35±15.65 | 58.84±14.92 | 0.764 |
| BMI (kg/m2) | 25.01±4.07 | 24.78±3.93 | 0.231 |
| Gender ,n％ |  |  | ＜0.001 |
| Male | 172（52.28） | 36（29.75） |  |
| Female | 157（47.72） | 85（70.25） |  |
| Hypertension, n％ |  |  | 0.716 |
| Yes | 101（30.70） | 35（28.93） |  |
| No | 228（69.30） | 86（71.07） |  |
| Diabetes mellitus, n％ |  |  | 0.008 |
| Yes | 98（29.79） | 21（17.36） |  |
| No | 231（70.21） | 100（82.64） |  |
| Coronary heart disease, n％ |  |  | 0.524 |
| Yes | 16（4.86） | 8（6.61） |  |
| No | 313（95.14） | 113（93.39） |  |
| Diameter of the calculus（cm） | 2.04±0.85 | 1.95±0.84 | 0.340 |
| Operation time（min） | 67.26±31.03 | 68.34±31.00 | 0.743 |
| Postoperative residual calculus, n％ |  |  | 0.242 |
| Yes | 7（2.13） | 5（4.13） |  |
| No | 322（97.87） | 116（95.87） |  |
| Postoperative indwelling double-J ureteral stent, n％ |  |  | 0.910 |
| Yes | 202（61.40） | 75（61.98） |  |
| No | 127（38.60） | 46（38.02） |  |
| Urinary leukocytes, n％ |  |  | 0.010 |
| Negative | 112（34.04） | 26（21.49） |  |
| Positive | 217（65.96） | 95（78.51） |  |
| Urinary protein, n％ |  |  | 0.347 |
| Negative | 209（63.53） | 71（58.68） |  |
| Positive | 120（36.47） | 50（41.32） |  |
| Urinary glucose, n％ |  |  | 0.330 |
| Negative | 270（82.07） | 104（85.95） |  |
| Positive | 59（17.93） | 17（14.05） |  |
| Urinary nitrite, n％ |  |  | 0.024 |
| Negative | 245（74.47） | 77（63.64） |  |
| Positive | 84（25.53） | 44（36.36） |  |
| Catheterization ,n％ |  |  | 0.268 |
| Yes | 147（44.68） | 47（38.84） |  |
| No | 182（55.32） | 74（61.16） |  |
| Hydronephrosis, n％ |  |  | 0.900 |
| Yes | 78（23.71） | 28（23.14） |  |
| No | 251（76.29） | 93（76.86） |  |
| Urogenital tumor ,n％ |  |  | 0.123 |
| Yes | 16（4.86） | 2（1.65） |  |
| No | 313（95.14） | 119（98.35） |  |
| Ureteral stricture ,n％ |  |  | 0.873 |
| Yes | 62（18.85） | 22（18.18） |  |
| No | 267（81.15） | 99（81.82） |  |
| Preoperative antibiotic use, n％ |  |  | 0.773 |
| Yes | 56（17.02） | 22（18.18） |  |
| No | 273（82.98） | 99（81.82） |  |
